# Supplementary material for: The use of bat houses as day roosts in macadamia orchards, South Africa
Source: PeerJ. 2019 May 22;7:e6954. doi: 10.7717/peerj.6954 (PMC6535037; doi:10.7717/peerj.6954)
Supplement: Dataset S3 [file peerj-07-6954-s003.docx]

**S1** Measurement (cm) and details of the bat houses in the study area Levubu, Limpopo, South Africa

| **Bathouse** | 4-Chamber | Old George | 6-Chamber | Rocket Box* | White&Black | Colony house |
| --- | --- | --- | --- | --- | --- | --- |
|  | Set of three | | |  | Set of two |  |
| **Number of sets/houses** | 7 | | | 5 | 2 | 1 |
| Height (cm) | 60 | 60 | 60 | 60 | 61 | 60 |
| Length (cm) | 50 | 50 | 28 | 20 | 43 | 90 |
| Width (cm) | 18 | 18 | 30 | 20 | 20 | 30 |
| Inner chamber(s) (cm) | 2 | 3 | 2 | 2 | 4.5 | 3 |
| Outer chamber (cm) | 3 | *Slanted* | 4 | 4.5 | - | - |
|  |  |  |  |  |  |  |
| **Landing pad** |  |  |  |  |  |  |
| Length (cm) | 50 | 50 | 28 | NA | 43 | NA |
| Height (cm) | 20 | 20 | 15 | NA | 15 | NA |
| Date erected/maintained | End of 2014/May 2016 | | | May 2016 | March 2016 | March 2016 |

**S2** All models, including the null and final model, testing the relationship between the occupancy of bat houses and the different variables in macadamia orchards, Levubu, South Africa, presented with AICc scores and delta AICc.

| **Model (Glmer)** | **delta** | **AICc** |
| --- | --- | --- |
| **Null model**  (Bat presence ~ (1\|Farm) + (1\|Month)) | 24.17 | 388.2 |
| Full Model  (Bat presence ~ Type of bat house + Altitude + Distance to Water +  Height of Bat House + (1\|Farm) + (1\|Month)) | 3.41 | 367.5 |
| Model 1  (Bat presence ~ Type of bat house + Altitude + (1\|Farm) + (1\|Month)) | 0.00 | 364.1 |
| **Model 2 (Final model)**  (Bat presence ~ Type of bat house + (1\|Farm) + (1\|Month)) | **0.50** | **364.6** |
| Model 3  (Bat presence ~ Type of bat house + Altitude + Height of Bat House + (1\|Farm) + (1\|Month)) | 1.39 | 365.5 |
| Model 4  (Bat presence ~ Type of bat house + Altitude + Distance to Water + (1\|Farm) + (1\|Month)) | 1.66 | 365.7 |
| Model 5  (Bat presence ~ Type of bat house + Distance to Water + (1\|Farm) + (1\|Month)) | 1.86 | 365.9 |

**S3** Bat species recorded during the study of bat houses on macadamia orchards, Levubu, Limpopo, South Africa

| **Farm** | **Month** | **Typ** | **Bat house** | **Location** | **Bats** | **Species** | **Cardinal** | **Altitude** | **Water** | **Height** |
| --- | --- | --- | --- | --- | --- | --- | --- | --- | --- | --- |
| JR | January | Bank | Nursery | Banana | 1 | Free-tailed bats | NE | 793 | 67 | 4 |
| DP | January | Set | Black | Pond | 1 | Mauritian tomb bat | SW | 754 | 2 | 3.5 |
| AW | January | Set | White | Hill | 1 | small Plain-faced bat | N | 932 | 680 | 5 |
| Amana | January | Bank | 6-Chamber | Site 2 | 3 | Yellow-bellied house bats | SW | 646 | 85 | 5 |
| AS | January | Bank | 6-Chamber | Dam | 4 | Yellow-bellied house bats | NE | 805 | 3 | 2.3 |
| AW | January | Set | Black | Hill | 3 | Yellow-bellied house bats | N | 932 | 680 | 5 |
| JR | February | Bank | Nursery | Banana | 1 | Free-tailed bats | NE | 793 | 67 | 4 |
| HJ | February | Bank | 6-Chamber | Old Shed | 5 | Yellow-bellied house bats | SW | 634 | 412 | 4 |
| AS | February | Bank | 6-Chamber | Dam | 4 | Yellow-bellied house bats | NE | 805 | 3 | 2.3 |
| DP | February | Set | Black | Pond | 4 | Yellow-bellied house bats | SW | 754 | 2 | 3.5 |
| AW | March | Set | White | Hill | 1 | Free-tailed bats | N | 932 | 680 | 5 |
| Amana | March | Bank | Old George | Site 2 | 1 | Yellow-bellied house bats | SW | 646 | 85 | 5 |
| HJ | March | Bank | Nursery | Old Shed | 2 | Yellow-bellied house bats | SW | 634 | 412 | 4 |
| AS | March | Bank | 6-Chamber | Dam | 3 | Yellow-bellied house bats | NE | 805 | 3 | 2.3 |
| AS | March | Bank | Nursery | Dam | 1 | Yellow-bellied house bats | NE | 805 | 3 | 2.3 |
| DP | March | Set | Black | Pond | 4 | Yellow-bellied house bats | SW | 754 | 2 | 3.5 |
| AW | March | Set | Black | Hill | 4 | Yellow-bellied house bats | N | 932 | 680 | 5 |
| JR | April | Bank | Nursery | Banana | 2 | Free-tailed bats | NE | 793 | 67 | 4 |
| Amana | April | Rocket Box | Rocket box | Dam | 2 | Yellow-bellied house bats | All | 646 | 155 | 5 |
| Amana | April | Bank | 6-Chamber | Site 2 | 4 | Yellow-bellied house bats | SW | 646 | 85 | 5 |
| HJ | April | Rocket Box | Rocket box | Site 1 | 1 | Yellow-bellied house bats | All | 607 | 165 | 4 |
| HJ | April | Bank | Nursery | Old Shed | 2 | Yellow-bellied house bats | SW | 634 | 412 | 4 |
| DP | April | Set | White | Pond | 1 | Yellow-bellied house bats | SW | 754 | 2 | 3.5 |
| JR | May | Bank | Nursery | Banana | 1 | Free-tailed bats | NE | 793 | 67 | 4 |
| HJ | May | Bank | Nursery | Old Shed | 1 | Free-tailed bats | SW | 634 | 412 | 4 |
| AW | May | Set | Black | Hill | 1 | Free-tailed bats | N | 932 | 680 | 5 |
| **Farm** | **Month** | **Typ** | **Bat house** | **Location** | **Bats** | **Species** | **Cardinal** | **Altitude** | **Water** | **Height** |
| JR | May | Bank | 6-Chamber | Dam | 1 | small Plain-faced bat | W | 772 | 37 | 4 |
| JR | May | Bank | 6-Chamber | Banana | 1 | small Plain-faced bat | NE | 793 | 67 | 4 |
| Amana | May | Rocket Box | Rocket box | Dam | 3 | Yellow-bellied house bats | All | 646 | 155 | 5 |
| Amana | May | Bank | 6-Chamber | Site 2 | 3 | Yellow-bellied house bats | SW | 646 | 85 | 5 |
| HJ | May | Rocket Box | Rocket box | Site 1 | 2 | Yellow-bellied house bats | All | 607 | 165 | 4 |
| AS | May | Bank | 6-Chamber | Dam | 3 | Yellow-bellied house bats | NE | 805 | 3 | 2.3 |
| AW | June | Set | Black | Hill | 1 | Free-tailed bats | N | 932 | 680 | 5 |
| JR | June | Bank | 6-Chamber | Banana | 2 | Free-tailed bats | NE | 793 | 67 | 4 |
| Amana | June | Bank | Nursery | Site 2 | 1 | Free-tailed bats | SW | 646 | 85 | 5 |
| AW | June | Set | White | Hill | 1 | Free-tailed bats | N | 932 | 680 | 5 |
| JR | June | Bank | Nursery | Dam | 1 | small Plain-faced bat | W | 772 | 37 | 4 |
| DP | June | Set | Black | Pond | 3 | small Plain-faced bat | SW | 754 | 2 | 3.5 |
| Amana | June | Bank | Nursery | Dam | 3 | Yellow-bellied house bats | NE | 646 | 46 | 5 |
| Amana | June | Bank | 6-Chamber | Site 2 | 5 | Yellow-bellied house bats | SW | 646 | 85 | 5 |
| AS | June | Bank | 6-Chamber | Dam | 1 | Yellow-bellied house bats | NE | 805 | 3 | 2.3 |
| Amana | June | Bank | 6-Chamber | Site 2 | 4 | Yellow-bellied house bats | SW | 646 | 85 | 5 |
| AS | June | Bank | 6-Chamber | Dam | 3 | Yellow-bellied house bats | NE | 805 | 3 | 2.3 |
| JR | July | Bank | 6-Chamber | Banana | 2 | Free-tailed bats | NE | 793 | 67 | 4 |
| DP | July | Set | Black | Pond | 1 | Free-tailed bats | SW | 754 | 2 | 3.5 |
| AW | July | Set | Black | Hill | 1 | Free-tailed bats | N | 932 | 680 | 5 |
| Amana | July | Bank | 6-Chamber | Site 2 | 1 | small Plain-faced bat | SW | 646 | 85 | 5 |
| Amana | July | Bank | 6-Chamber | Dam | 4 | Yellow-bellied house bats | NE | 646 | 46 | 5 |
| Amana | July | Bank | Old George | Site 2 | 3 | Yellow-bellied house bats | SW | 646 | 85 | 5 |
| AS | July | Bank | 6-Chamber | Dam | 2 | Yellow-bellied house bats | NE | 805 | 3 | 2.3 |
| Amana | July | Bank | 6-Chamber | Site 2 | 3 | Yellow-bellied house bats | SW | 646 | 85 | 5 |
| AS | July | Bank | 6-Chamber | Dam | 3 | Yellow-bellied house bats | NE | 805 | 3 | 2.3 |
| JR | August | Bank | 6-Chamber | Banana | 1 | Free-tailed bats | NE | 793 | 67 | 4 |
| Amana | August | Bank | 6-Chamber | Dam | 1 | Free-tailed bats | NE | 646 | 46 | 5 |
| **Farm** | **Month** | **Typ** | **Bat house** | **Location** | **Bats** | **Species** | **Cardinal** | **Altitude** | **Water** | **Height** |
| AW | August | Set | Black | Hill | 1 | Free-tailed bats | N | 932 | 680 | 5 |
| Amana | August | Bank | Old George | Site 2 | 2 | small Plain-faced bat | SW | 646 | 85 | 5 |
| HJ | August | Bank | Old George | Site 1 | 3 | Yellow-bellied house bats | S | 607 | 160 | 4 |
| AS | August | Bank | 6-Chamber | Dam | 1 | Yellow-bellied house bats | NE | 805 | 3 | 2.3 |
| AW | September | Set | Black | Hill | 2 | Free-tailed bats/  small Plain-faced bat | N | 932 | 680 | 5 |
| Amana | September | Bank | 6-Chamber | Dam | 4 | Yellow-bellied house bats | NE | 646 | 46 | 5 |
| HJ | September | Bank | Nursery | Site 1 | 4 | Yellow-bellied house bats | S | 607 | 160 | 4 |
| JR | October | Bank | Nursery | Banana | 2 | small Plain-faced bat | NE | 793 | 67 | 4 |
| JR | October | Bank | Old George | Dam | 1 | Yellow-bellied house bats | W | 772 | 37 | 4 |
| Amana | October | Bank | Old George | Dam | 1 | Yellow-bellied house bats | NE | 646 | 46 | 5 |
| Amana | October | Bank | 6-Chamber | Site 2 | 3 | Yellow-bellied house bats | SW | 646 | 85 | 5 |
| HJ | October | Bank | Old George | Site 1 | 3 | Yellow-bellied house bats | S | 607 | 160 | 4 |
| JR | November | Bank | 6-Chamber | Banana | 3 | Free-tailed bats | NE | 793 | 67 | 4 |
| Amana | November | Bank | 6-Chamber | Site 2 | 2 | Yellow-bellied house bats | SW | 646 | 85 | 5 |
| HJ | November | Bank | Old George | Site 1 | 4 | Yellow-bellied house bats | S | 607 | 160 | 4 |
| JR | December | Bank | Nursery | Banana | 2 | Free-tailed bats | NE | 793 | 67 | 4 |
| AW | December | Set | Black | Hill | 1 | Free-tailed bats | N | 932 | 680 | 5 |
| HJ | December | Bank | Old George | Site 1 | 4 | Yellow-bellied house bats/  Mauritian tomb bat | S | 607 | 160 | 4 |
| AS | December | Bank | 6-Chamber | Dam | 5 | Yellow-bellied house bats/  Mauritian tomb bat | NE | 805 | 3 | 2.3 |
